# Deuterium in marine organic biomarkers: toward a new tool for quantifying aquatic mixotrophy

**DOI:** 10.1111/nph.18023

**Published:** 2022-03-04

**Authors:** Marc‐André Cormier, Jean‐Baptiste Berard, Gaël Bougaran, Clive N. Trueman, Daniel J. Mayor, Richard S. Lampitt, Nicholas J. Kruger, Kevin J. Flynn, Rosalind E. M. Rickaby

**Affiliations:** ^1^ 6396 Department of Earth Sciences University of Oxford South Parks Road Oxford OX1 3AN UK; ^2^ IFREMER, Physiology and Biotechnology of Algae (PBA) Laboratory rue de l'Ile d'Yeu, BP 21105 Nantes Cedex 3 44311 France; ^3^ Ocean and Earth Science National Oceanography Centre Southampton University of Southampton Southampton SO14 3ZH UK; ^4^ Ocean Biogeosciences National Oceanography Centre Southampton SO14 3ZH UK; ^5^ 6396 Department of Plant Sciences University of Oxford South Parks Road Oxford OX1 3RB UK; ^6^ 61564 Plymouth Marine Laboratory Prospect Place Plymouth PL1 3DH UK

**Keywords:** biomarker, carbon cycle, hydrogen, isotope, mixoplankton, mixotrophy, protist

## Abstract

The traditional separation between primary producers (autotrophs) and consumers (heterotrophs) at the base of the marine food web is being increasingly replaced by the paradigm that mixoplankton, planktonic protists with the nutritional ability to use both phago(hetero)trophy and photo(auto)trophy to access energy are widespread globally. Thus, many ‘phytoplankton’ eat, while 50% of ‘protozooplankton’ also perform photosynthesis. Mixotrophy may enhance primary production, biomass transfer to higher trophic levels and the efficiency of the biological pump to sequester atmospheric CO_2_ into the deep ocean. Although this view is gaining traction, science lacks a tool to quantify the relative contributions of autotrophy and heterotrophy in planktonic protists. This hinders our understanding of their impacts on carbon cycling within marine pelagic ecosystems. It has been shown that the hydrogen (H) isotopic signature of lipids is uniquely sensitive to heterotrophy relative to autotrophy in plants and bacteria. Here, we explored whether it is also sensitive to the trophic status in protists. The new understanding of H isotope signature of lipid biomarkers suggests it offers great potential as a novel tool for quantifying the prevalence of mixotrophy in diverse marine microorganisms and thus for investigating the implications of the ‘mixoplankton’ paradigm.

## Introduction

**Fig. 1:**
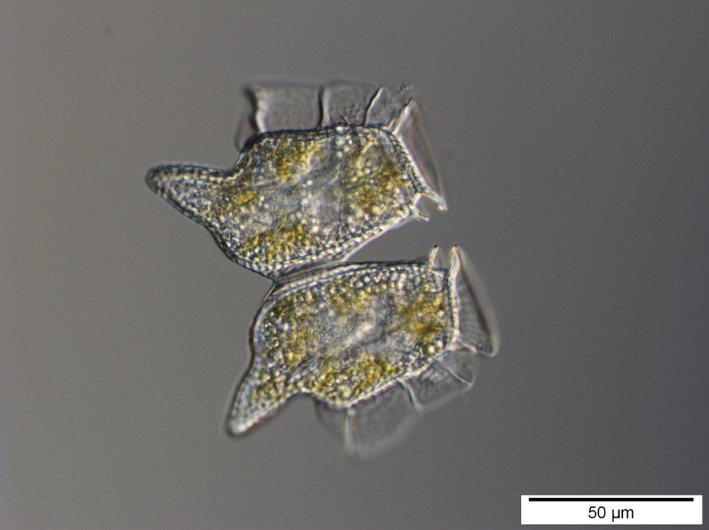
Mixoplanktonic dinoflagellate *Dinophysis caudata*. The concept of animal and vegetable gets blurred when applied to unicellular organisms. Dinoflagellates can perform photosynthesis while devouring prey by phagocytosis and consuming dissolved organic molecules via osmotrophy. This latter mechanism is ubiquitous in phytoplankton. Photo by Nicolas Chomérat & Véronique Séchet (Ifremer).

Marine ecosystems play a pivotal role in global photosynthetic carbon fixation (Field *et al*., 1998; Falkowski *et al*., 2000). Their activity contributes to maintaining the balance between O_2_ and CO_2_ in the atmosphere and consequently to keeping climate relatively stable. The biological gravitational pump exports between *c.* 4.0 and 9.1 Pg of particulate organic carbon from surface waters annually (Boyd *et al*., 2019). Most current marine biogeochemical models assume that the plankton community is clearly divided into autotrophic phytoplankton and heterotrophic zooplankton (Duarte *et al*., 2013; Flynn *et al*., 2013; Williams *et al*., 2013; Leles *et al*., 2018). Increasingly, it is recognised that there is no strict separation between producers and consumers (Fig. 1) and that photo(auto)trophic and phago(hetero)trophic behaviours are not mutually exclusive. Indeed, most of the protist unicellular organisms at the base of the plankton food web cannot be regarded strictly as producers or consumers (Flynn *et al*., 2013, 2019; Mitra *et al*., 2014). Modelling suggests that mixoplanktonic activity (i.e. nutrition involving both autotrophy and phagotrophy) enhances primary production, biomass transfer to higher trophic levels and the biological carbon pump by up to 35% (Mitra *et al*., 2014; Ward & Follows, 2016; Leles *et al*., 2021). To this, we add the renewed interest in mixotrophy supported by a combination of phototrophy and osmotrophy in diatoms and also in oceanic prokaryote phytoplankton (e.g. Yelton *et al*., 2016; Benavides *et al*., 2017; Muñoz‐Marín *et al*., 2020). The mixoplanktonic behaviour of many toxic protists also likely explains their ecological success and the occurrence of harmful algal blooms (HABs) (Burkholder *et al*., 2008) that can severely affect coastal ecosystems and their services. Changing the prevalence of mixotrophic behaviours (involving osmotrophy and/or phagotrophy) within the plankton community could have a large impact on the global carbon cycle and thus on the climate. For example, a large >50‐yr time series analysis suggests an increase in the relative abundance of diatoms vs dinoflagellates (Hinder *et al*., 2012). Another example, using quantitative niche models, suggests that oceanic cyanobacterial communities will experience complex changes as a result of projected future climate conditions (Flombaum *et al*., 2013). These changes may result in different mixotrophic activities in the surface ocean and affect the efficiency of the biological pump. Thus, there are a number of reasons for having a better understanding of the role of mixotrophy in marine microbial ecology.

Even though mixoplankton (i.e. photosynthetic microbes capable of phagotrophy or ‘photo‐osmo‐phago‐trophic’ plankton) are a key component of marine ecosystems (Leles *et al*., 2017, 2019), with all other phytoplankton capable of mixotrophy via osmotrophy (Flynn *et al*., 2019), no tool currently exists to infer, both spatially and temporally, the trophic mode and the level of heterotrophic growth within different plankton groups. Tracking protist trophic modes, without an appropriate tool, in modern oceans is challenging as their species‐specific metabolism and trophic adaptability remain largely unknown. Tracking their behaviour over geological time presents even greater challenges. For example, it was recently suggested that protists, mainly haptophytes, ‘turned to hunting’ in order to survive the end‐Cretaceous impact (Gibbs *et al*., 2020). However, due to the absence of any direct proxy, only indirect lines of evidence, such as eco‐evolutionary modelling and microfossils, are available to support this hypothesis. Only a fraction of protist taxa produce an observable microfossil in sediments (e.g. Radi *et al*., 2013; Cormier *et al*., 2016; Sophie *et al*., 2021). Such difficulties, combined with the need for data to better constrain biogeochemical models, drive the need for a means of tracking protists’ metabolic behaviour (particularly the prevalence of mixoplanktonic lifestyle) in response to global environmental changes (Chavez *et al*., 2011). This would facilitate the incorporation of mixotrophy into marine ecosystem models that are used to examine the future effects of anthropogenic perturbation on biogeochemical cycling in the global ocean.

## Stable isotope ratios

Variations in the relative abundance of stable isotopes of diverse elements (e.g. hydrogen, carbon, nitrogen and oxygen) give important information about (bio)geochemistry and palaeoclimate. The measurement of their relative abundance, as preserved in geological and biological archives (e.g. sediment cores, tree rings, plankton and herbaria), is ubiquitous over a broad range of earth science studies. Generally expressed with a delta notation (Werner & Cormier, 2022), much of what we know about the past functioning of the biosphere comes from these measurements.

Isotopes of an element have identical chemical properties because the chemical reactivity is determined by the valence electrons. However, they do not behave like identical twins due to the differences in atomic mass, which lead to isotopic fractionation in their abundance during chemical and/or biological reactions (often termed α or ε). The rate of kinetic reactions and the position of chemical equilibria can be different when different isotopes are involved. Different nuclear masses will influence the intramolecular bond energies due to the lower vibrational energy when a heavier isotope is substituted on the bond. Molecules in which positions are substituted with heavy isotopes are more stable, and more energy is needed to cleave a covalent bond between atoms when heavier isotopes are involved. Correspondingly, a greater amount of energy is released during bond formation with heavier isotopes at equilibrium (Werner & Cormier, 2022). Light isotopes react more quickly than heavy isotopes due to their lower activation energy during reactions influenced by kinetics.

There are indications that the relative abundance of hydrogen (H) isotopes in lipids (i.e. deuterium (^2^H) and protium (^1^H), expressed as δ^2^H values) is highly sensitive to the metabolism of bacteria (Zhang *et al*., 2009; Wijker *et al*., 2019), vascular plants (Luo & Sternberg, 1992; Cormier *et al*., 2018), in particular orchids (Gebauer *et al*., 2016; Schiebold *et al*., 2018), and parasitic plants (Cormier *et al*., 2019). Despite these indications, the application of the ^2^H content in organic biomarkers has been largely limited to (palaeo)hydrological or salinity studies. Indeed, there have been various studies focused on using δ^2^H values of organic material from terrestrial plants to reconstruct hydrology (Sachse *et al*., 2012), and a handful of studies on marine phototrophs, with a focus almost entirely on the effects of salinity on lipid δ^2^H values (Kasper *et al*., 2014). Nonetheless, the effect of metabolism on δ^2^H values provides evidence that δ^2^H values can offer other valuable biogeochemical information (Estep & Hoering, 1980; Ziegler, 1989; Luo & Sternberg, 1992; Zhang *et al*., 2009; Meer *et al*., 2015; Gebauer *et al*., 2016; Cormier *et al*., 2018, 2019). Here, we suggest that δ^2^H values of marine organic material could become a useful tool to study the role of mixotrophy in marine ecosystems.

Two main factors determine the δ^2^H values of organic compounds produced by aquatic organisms: (1) the δ^2^H value of the source water (Chikaraishi & Naraoka, 2003); and (2) the biosynthetic ^2^H fractionation (^2^H‐ε_bio_) (Sternberg *et al*., 1984; Yakir & DeNiro, 1990; Luo *et al*., 1991; Zhang & Sachs, 2007). The latter involves several biochemical pathways and is calculated as the ^2^H fractionation between water and the synthesised organic compounds. Because of the strength of the C‐H bond in lipids, δ^2^H values of lipids are relatively stable over time. Isotopic exchanges of C‐bound H can nevertheless slowly occur over geological timescales. Such exchange can be recognised (e.g. via comparison of coeval n‐alkyl and isoprenoid hydrocarbons) and does not preclude the valuable use of δ^2^H values in geological studies (Sessions, 2016). Most palaeostudies using δ^2^H derived from terrestrial plant biomarkers have considered ^2^H‐ε_bio_ to be constant within a species (Sachse *et al*., 2006) simplifying the application of biomarker δ^2^H values as a (palaeo)hydrological proxy (Rach *et al*., 2014, 2017; Ladd, 2021). In plankton, however, salinity and growth rate are also known to influence ^2^H‐ε_bio_ during lipid biosynthesis (Englebrecht & Sachs, 2005; Schouten *et al*., 2006). It is mainly because of the influence of salinity on ^2^H‐ε_bio_ that δ^2^H values can be used to derive palaeosalinity in aquatic basins (Ladd & Sachs, 2015). Evaporation in (semi‐)closed aquatic basins also influences both salinity and source water δ^2^H values, which is correspondingly recorded in the δ^2^H values of the biomarkers produced in those basins (Nelson & Sachs, 2016).

Most models of δ^2^H values in organic compounds have endeavoured to minimise the impact of metabolism in order to explain the variability in δ^2^H values as a result of a specific environmental parameter. Roden *et al*. (2000) suggested that in plants, ^2^H‐depleting photosynthetic fractionation pathways and ^2^H‐enriching post‐photosynthetic fractionation pathways determine a constant value for ^2^H‐ε_bio_. While this approach is very useful for interpreting hydrological conditions from cellulose in tree rings, it does not capture the effects of environmental change on the balance between photosynthetic and post‐photosynthetic processes and their impact on δ^2^H values. Additionally, the approach has not yet been extended to compounds other than cellulose or to taxa other than angiosperms.

## A new conceptual view of H isotope ratios

To fill these gaps, a conceptual biochemical model has been proposed to describe how post‐photosynthetic processes imprint a strong metabolic signal in δ^2^H values of plant‐derived organic compounds (i.e. up to 60‰) in response to environmental changes (Cormier *et al*., 2018, 2019). These biochemical pathways leave a metabolic signal on the δ^2^H values because they induce a different isotopic fractionation. The model expresses that, overall, photosynthetic pathways deplete organic compounds in ^2^H, while post‐photosynthetic (or heterotrophic) pathways enrich compounds in ^2^H.

**Fig. 2:**
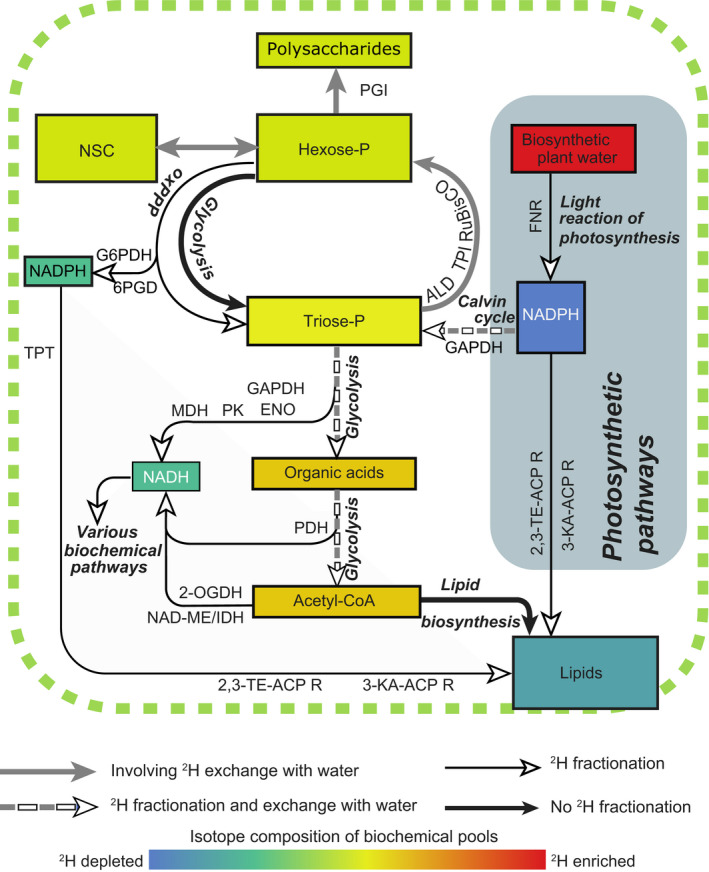
Schematic view of H flow during processes leading to lipids, polysaccharides and nonstructural carbohydrates (NSC) ^2^H‐ε_bio_. The key enzymes and pathways responsible for H flow are indicated by their following abbreviations and are based on known biochemical pathways: 2‐OGDH, 2‐oxoglutarate dehydrogenase; 6PGD, 6‐phosphogluconate dehydrogenase; ACP, acyl carrier protein; ALD, aldolase; ENO, enolase; FNR, ferredoxin‐NADP^+^ reductase; G6PDH, glucose‐6‐phosphate dehydrogenase; GAPDH, glyceraldehyde 3‐phosphate dehydrogenase; KA, ketoacyl; ME, malic enzyme; NADP, nicotinamide adenine dinucleotide; NADPH, nicotinamide adenine dinucleotide phosphate; oxPPP, oxidative pentose phosphate pathway; PDH, pyruvate dehydrogenase; PGI, phosphoglucose isomerase; PK, pyruvate kinase; R, reductase; RuBisCO, ribulose‐1,5‐bisphosphate carboxylase/oxygenase; TE, *trans*‐enoyl; TPI, triosephosphate isomerase; and TPT, triose phosphate translocator. Simplified from Cormier *et al*. (2018) and references therein.

**Fig. 3:**
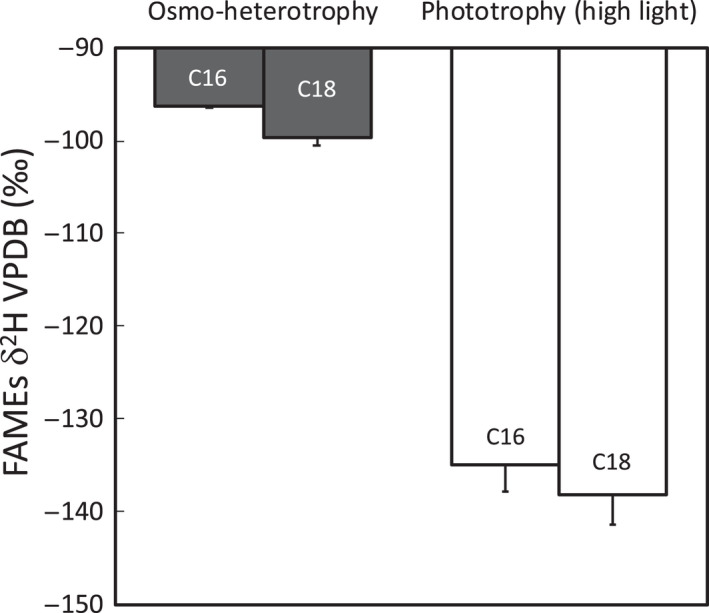
Average δ^2^H values of fatty acids (methyl esterified) produced by *Chlorella sorokiniana* under different metabolic conditions. For the heterotrophic mode, *C. sorokiniana* was fed with glucose in the dark. C16 and C18 correspond to palmitic and stearic acid, respectively. Error bars correspond to ± SD (*n* = 3).

Specifically, in Cormier's model, the photosynthetic carbohydrate supply rate affects ^2^H‐ε_bio_ for carbohydrates and lipids (Fig. 2). This pattern is mostly driven by the carbohydrate pool size, the cycling rates of individual organic molecules in their respective pools, the associated exchange of C‐bound H with ^2^H‐enriched cellular water and preferential removal of light ^1^H via the oxidative pentose phosphate pathway (oxPPP) (Cormier *et al*., 2018). Consequently, a high photosynthetic (e.g. autotrophically dominated) carbon supply results in ^2^H‐depleted organic molecules, reflecting the ^2^H‐depleted signal of NADPH generated by the light reaction of photosynthesis (i.e. grey zone; Fig. 2). By contrast, a low photosynthetic (e.g. heterotrophically dominated) carbon supply leads to ^2^H‐enriched lipids, where higher cycling rates of individual organic molecules are associated with an increasing exchange of C‐bound H with ^2^H‐enriched cellular water and removal of ^1^H from the C‐skeletons via the oxPPP. While such a model highlights the likely considerable effect of fluxes through metabolic networks on H isotopic fractionation (Kruger & Ratcliffe, 2015), it also explains why a metabolic signal in H isotopic fractionation due to a shift in trophic behaviour is discernible in diverse biological organisms. As such, Gebauer *et al*. (2016) recently adopted this rationale successfully for analysis of environmental samples to study orchid ecology and the prevalence of heterotrophic and mycoheterotrophic orchid taxa in Europe. Similar to most marine protists, orchids behave auto‐, mixo‐ and heterotrophically. The successful application of δ^2^H values to study orchid ecology underlines the potential of compound‐specific δ^2^H values as a metabolic proxy in diverse environments and biological systems. In plants, studying such biochemical effects is greatly complicated by the spatial and temporal variation of the isotopic composition of water, the H source (i.e. within soil–water gradients and throughout plant stems and leaves). By contrast, sea water has an essentially constant δ^2^H value in comparison with the scale of the biological fractionations, allowing a primary focus on biochemical variations in plankton. In preliminary work (Supporting Information Notes S1), we have observed that hydrogen isotopic fractionation during fatty acid biosynthesis in the green algae *Chlorella sorokiniana* is also sensitive to heterotrophy (Fig. 3). This metabolic sensitivity represents a fundamentally different philosophical approach to most previous studies of ^2^H fractionation in photo(auto)trophs, which have assumed near‐constant biochemistry and sought to reconstruct environmental variables.

## The way towards the new tool

**Fig. 4:**
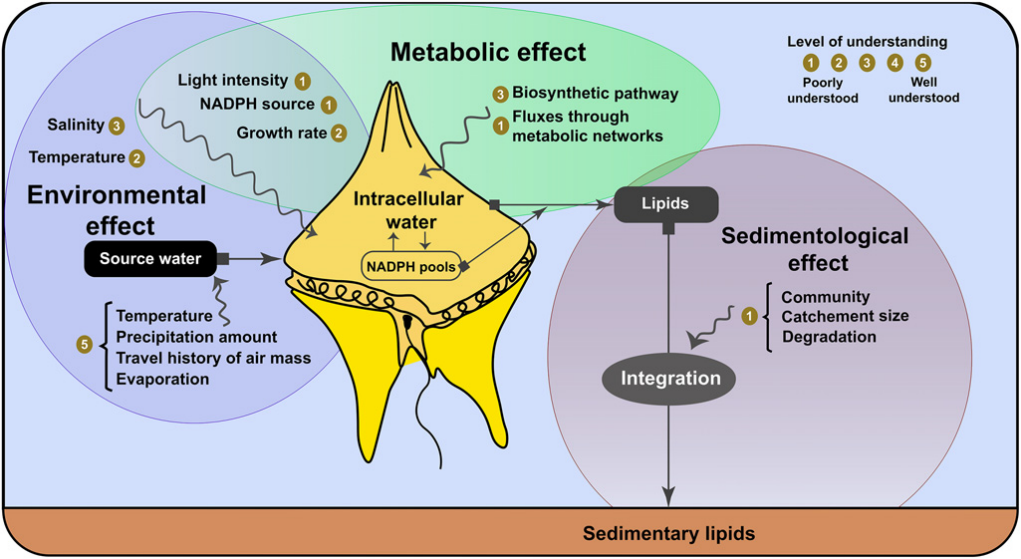
Overview of the processes affecting δ^2^H values of lipid biomarkers in protists, where they take place (wiggly arrows), and their current level of understanding (inspired by Sachse *et al*., 2012). To effectively use δ^2^H values as a tool to quantify mixotrophic processes, key limitations in their understanding need to be resolved.

Laboratory‐based calibrations are essential for *in situ* monitoring of protist trophic behaviours (including phago(hetero)trophic vs photo(auto)trophic growth) using isotopic measurements of lipid biomarkers from environmental samples. For instance, the magnitude of the photosynthetic ^2^H fractionation, which occurs during the light reaction of the photosynthesis (Fig. 2, grey zone), has only been estimated by Yakir & DeNiro (1990) who reported a fractionation of −171‰ for cellulose in the multicellular aquatic plant *Lemna gibba*. This value, although widely utilised, most likely varies appreciably between organisms and trophic behaviours. Moreover, even if some variables are known to influence post‐photosynthetic ^2^H fractionation during lipid biosynthesis (e.g. temperature, salinity, light intensity, growth rate, biosynthetic pathway, metabolic network and metabolic source of NADPH), a comprehensive understanding of how these variables impact, individually and synergistically, on ^2^H fractionation in marine microbes is still lacking (Fig. 4).

With the appropriate investigations targeting these variables, compound‐specific isotope analysis has the potential to provide a valuable research tool: a metabolic proxy for the quantitative assessment of the ratio between autotrophic and heterotrophic metabolism in diverse marine microorganisms and their contribution to the global carbon cycle in modern and, potentially, palaeoenvironmental contexts (i.e. assuming that the metabolic effects can be deconvoluted from the environmental and sedimentological effects using other proxies). Moreover, defining the metabolic influences on H isotope fractionation during biomarker synthesis will provide a much better understanding of their δ^2^H values in response to changes in environmental conditions and will improve their utility as versatile palaeoecological proxies for, *inter alia*, temperature (Feng & Epstein, 1994), hydrological conditions (Sachse *et al*., 2012) and sea surface salinity (Kasper *et al*., 2014).

## Author contributions

M‐AC, REMR and NJK planned and designed the research. M‐AC, J‐BB and GB designed and performed the experiment and analysed the data. M‐AC and J‐BB performed chemical measurements. M‐AC, KJF and REMR wrote the manuscript with contributions from CNT, DJM and RSL.

## Supporting information


**Notes S1** Cultures of *Chlorella sorokiniana* and compound‐specific isotope analyses.
Please note: Wiley‐Blackwell are not responsible for the content or functionality of any Supporting Information supplied by the authors. Any queries (other than missing material) should be directed to the *New Phytologist* Central Office.

## Data Availability

The data that support the findings of this study are available in Supporting Information Notes S1.
