# Supplementary material for: Deuterium in marine organic biomarkers: toward a new tool for quantifying aquatic mixotrophy
Source: New Phytol. 2022 Mar 4;234(3):776–82. doi: 10.1111/nph.18023 (PMC9310953; doi:10.1111/nph.18023)
Supplement: Supplementary file 1 — Notes S1 Cultures of Chlorella sorokiniana and compound‐specific isotope analyses. Please note: Wiley‐Blackwell are not responsible for the content or functionality of any Supporting Information supplied by the authors. Any queries (other than missing material) should be directed to the New Phytologist Central Office. [file NPH-234-776-s001.pdf]

Notes S1: Cultures of *Chlorella sorokiniana* and Compound-Specific Isotope Analyses

Supporting Information for

"Deuterium in marine organic biomarkers: Toward a new tool for quantifying aquatic mixotrophy" accepted in *New Phytologist* on 3 February 2022.

by

Marc-André Cormier[a], Jean-Baptiste Berard[b], Gaël Bougaran[b], Clive Trueman[c], Daniel J. Mayor[d], Richard S. Lampitt[d], Nicholas J. Kruger[e], Kevin J. Flynn[f], Rosalind E. M. Rickaby[a].

Affiliations:

[a]University of Oxford, Department of Earth Sciences, South Parks Road, Oxford, OX1 3AN, UK
[b]IFREMER, Physiology and Biotechnology of Algae (PBA) Laboratory, rue de l'Ile d'Yeu, BP 21105, 44311 Nantes cedex 3, France
[c]Ocean and Earth Science, University of Southampton, Southampton, SO14 3ZH, UK
[d]Ocean Biogeosciences, National Oceanography Centre, Southampton, SO14 3ZH, UK
[e]University of Oxford, Department of Plant Sciences, South Parks Road, Oxford, OX1 3RB, UK
[f]Plymouth Marine Laboratory, Plymouth, Prospect Place, PL1 3DH, UK
[*]Corresponding author: ma_cormier@alumni.ethz.ch / +44 7488 702520

Cultures of *Chlorella sorokiniana*. An axenic strain of *Chlorella sorokiniana* SAG 211-31 was used for triplicate batch cultures under autotrophic (AC) and heterotrophic (HC) conditions. Autoclaved flasks containing 400 mL of 0.2 µm-filtered freshwater enriched with modified Walne's Medium (Walne, 1970) were used for both conditions. Modifications consisted in using ammonium (NH_4^+) instead of nitrate as the sole source of nitrogen. Potassium (20 µM) and calcium (0.7 µM) were also added to the medium. Glucose (55 mM) was added as organic carbon source for HC, and inorganic carbon input was supplied by an air bubbling for both HC and AC. This also provided airlifting stirring to the cultures. The medium was adjusted to pH 6.8 for both conditions using Trizma-acid and Trizma-base. Temperature was set at 25 °C. A photosynthetic photon fluence rate of 500 µmol m^{-2} s^{-1} over the waveband 400–700 nm was used for AC. The HC cultures were placed in complete darkness by wrapping the flasks in several layers of aluminium foil.

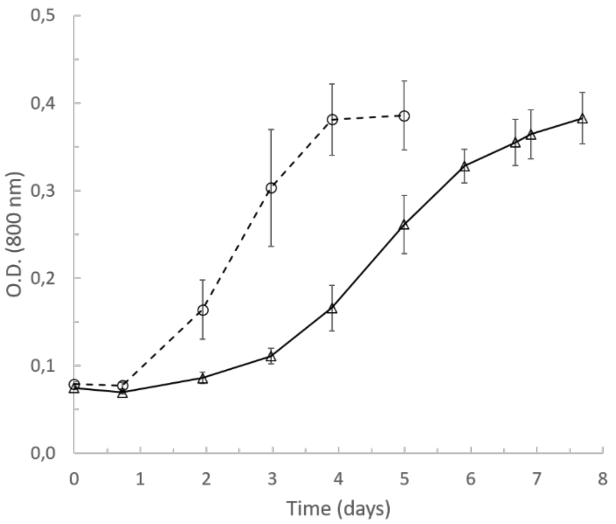

Supporting information Fig. S1. Growth characteristics of AC cultures (round dots and dashed lines) and HT cultures (triangle dots and solid lines). Growth was monitored by measuring optical density at 800 nm. The values are presented as the mean with error bars representing ± SD (n = 3).

Notes S1: Cultures of *Chlorella sorokiniana* and Compound-Specific Isotope Analyses

Monitoring. Cultures were monitored daily for growth using optical density measurements at 800 nm (Supporting information Fig. S1) and for axenicity using epifluorescence microscopy with SYBR Green staining (Lonza, USA). The availability of nitrogen was also checked by measuring residual extracellular NH_4^+ on the last sampling day (i.e., when cultures had reached stationary phase: 5 and 8 days for AC and HC, respectively) using an auto-analyser (Seal Analytical AA3) according to Ifremer's standardized protocol (Aminot & Kérouel, 2007). When the cultures were in stationary phase, NH_4^+ concentrations were below the limit of quantification (<0.05 µM).

Compound-Specific Isotope Analyses (CSIA). For complete details of lipid purification and preparation methods for CSIA, see Peters et al. (2005). All glassware used was pre-combusted at 450 °C. Briefly, the cultures were filtered on individual 47 mm GF/C filters. Prior to chemical purifications, the filters were frozen at -80°C and freeze-dried. The lipids (including fatty acids) were extracted from the filtrates in 7 mL glass vials. The filtrates were saponified with a mixture of CH_3OH : H_2O (9 : 1) and 5 % KOH at 70 °C for 1 h. Neutral lipids were separated from the mixture by liquid-liquid extraction with hexane. Subsequently, the remaining fatty acids, in the aqueous phase, where also separated from the mixture by liquid-liquid extraction with hexane after their reduction with 37 % HCl. Following their separation, fatty acids were methyl-esterified with a solution of 5 % HCl in CH_3OH, under at atmosphere of N_2, at 70°C overnight. Following derivatisation, fatty acids methyl-esters (FAMEs) and trace hydroxy-FAMEs were extracted from the mixture by liquid-liquid extraction with hexane. Finally, prior to analyses, the hydroxy-FAMEs were separated from the FAMEs by column chromatography by eluting 4 mL hexane in glass silica-gel columns. The columns were pre-prepared by filling about three-quarters of the column volume with 2 g silica-gel. The columns were rinsed with 10 mL acetone, 10 mL dichloromethane and 10 mL hexane and finally chemically activated in a desiccation oven at 60 °C overnight.

Following the method described by Sessions (2006), δ^2H values of FAMEs were measured at the University of Basel on a Delta V plus stable isotope ratio mass spectrometer (IRMS) coupled to a Trace gas chromatograph (GC) Ultra and a GC Isolink via a ConFlo IV (Thermo Fisher Scientific, USA). FAMEs of different chain-lengths were separated by GC using an Agilent (DB-5) 30 m column with a diameter of 0.250 mm and a film thickness of 0.25 µm. The injector was in spitless mode at a temperature of 270 °C. The GC oven temperature was held at 90 °C for 2 min, then raised to 150 °C at 10 °C per min, then to 320 °C at 4 °C per min. This final temperature was held for 10 min. Individual compounds were converted to H_2 gas in an aluminium oxide reactor (Thermo Fisher Scientific, USA) at 1420 °C and each sample was measured in duplicate. For isotope measurements, samples were dissolved in hexane at a concentration of about 300 ng of the most abundant FAMEs per µL of hexane, previously identified and quantified using a 7890A GC coupled with a flame ionization detector (FID, Agilent Technologies, USA) operating with the same GC method. Specific FAMEs quantifications were performed via comparison with α-androstane used as an internal standard.

Notes S1: Cultures of *Chlorella sorokiniana* and Compound-Specific Isotope Analyses

References

Aminot A, Kérouel R. **2007**. *Automatique des Nutriments dans les Eaux Marines: Méthodes en flux Continu*. Ifremer - QUAE.

Peters KE, Walters CC, Moldowan JM. **2005**. *The Biomarker Guide: Biomarkers and isotopes in the environment and human history*. Cambridge University Press.

Sessions AL. **2006**. Isotope-ratio detection for gas chromatography. *Journal of Separation Science* **29**: 1946 1961.

Walne R Peter. **1970**. *Studies on the Food Value of Nineteen Genera of Algae to Juvenile Bivalves of the Genera Ostrea, Crassostrea, Mercenaria and Mytilus*. London, UK: Her Majesty's Stationery Office.
